# Development and Application of Mouse-Derived CD2v Monoclonal Antibodies Against African Swine Fever Virus from Single B Cells

**DOI:** 10.3390/v17081123

**Published:** 2025-08-15

**Authors:** Litao Yu, Fangtao Li, Xingqi Zou, Lu Xu, Junjie Zhao, Yan Li, Guorui Peng, Yingju Xia, Qizu Zhao, Yuanyuan Zhu

**Affiliations:** National/WOAH Reference Laboratory for Classical Swine Fever, China Institute of Veterinary Drug Control, Beijing 100081, China

**Keywords:** African swine fever virus, single B-cell antibody technology, CD2v, blocking ELISA

## Abstract

African swine fever (ASF) is a highly pathogenic and hemorrhagic swine infectious disease caused by the African swine fever virus (ASFV). It encodes over 150 proteins, among which the CD2v protein plays multiple roles throughout the infection process. Single B-cell antibody technology is a cutting-edge method for preparing monoclonal antibodies (mAbs), which has the advantages of rapid, efficient, and high yield in antibody production, while possessing natural conformations. In this study, by cloning and expressing antibody genes in vitro, 14 murine-derived mAbs were prepared using recombinant CD2v proteins as immunogenic sources, which brings sufficient enrichment and selectivity for the development of antibodies based on the single B-cell antibody technique. All 14 mAbs demonstrated reactivity with CD2v protein by indirect ELISA, whereas 8 mAbs successfully detected CD2v in ASFV-infected PAM cells by IFA, indicating the tested mAbs can effectively recognize and bind to ASFV CD2v. Finally, a blocking ELISA method for detecting CD2v antibodies using CD2v mAb C89 was established, which holds significant potential for broad application in the serological diagnosis of ASFV with determination of the CD2v-blocking ELISA specificity, sensitivity, reproducibility, and compliance rate. It could be used for the rapid clinical detection of ASFV CD2v protein to provide a powerful tool for the monitoring of epidemics.

## 1. Introduction

African swine fever (ASF), which first emerged in China in 2018 [1], is an acute, virulent, and hemorrhagic swine infectious disease caused by African swine fever virus (ASFV). It has been listed as one of the notified diseases by the World Organization for Animal Health (WOAH) and as a class I animal disease in China [2]. Currently, ASF is still spreading widely, causing immeasurable losses to the pig industry worldwide. Vaccination is one of the most effective strategies to prevent and control infectious diseases. However, there are currently no commercial vaccines for ASF, so strict biosafety control and culling policies are mainly used to prevent and control this disease in China.

ASFV is the only member of the Asfarviridae family, genus Asfivirus, which is the only insect-borne DNA virus found to possess a complex double-stranded DNA structure, with mature virus particles in the form of symmetrical icosahedra. ASF involves domestic pigs, reservoir hosts in wildlife (wild boar or other feral swine), inanimate fomites (carcasses, contaminated habitats, tools, and other mechanical vectors), and competent arthropod vectors (soft ticks) [3]. ASFV can be transmitted by the Ornithodoros species of soft ticks, but these are not required for transmission between domestic pigs or wild boar [4]. The main ASFV vesicle membrane proteins with known functions are p72, CD2v, p12, p54, p30, p17, pE248R, pE183L, and pH108R. ASFV has been classified into 24 genotypes based on the nucleotide sequence of the B646L gene encoding the p72 protein, and into eight serotypes based on the EP402R gene encoding the CD2v protein [5,6]. CD2v is a transmembrane glycoprotein that shares two predicted immunoglobulin (Ig) superfamily (Ig SF) domains with host CD2 molecules [7], of which the N-terminal Ig-like domain was identified as the putative CD2v erythrocyte-binding area [8]. The CD2v protein is a characteristic viral protein of ASFV, which is capable of inducing protective immune responses and possesses the CD2v vesicle protein encoded by the ASFV EP402R gene and has various functions, including mediating hemocyte adsorption, inhibiting host cell immunoregulation, and participating in the induction of apoptosis and viral immune escape [9]. At present, several Mabs have been prepared using CD2v as an immunogen [10,11]. CD2v candidate antibodies for blocking ELISA were screened, while a blocking ELISA based on the CD2v protein was successfully established [12]. Since CD2v protein plays multiple roles in the whole infection process of ASFV, Mabs against CD2v protein are not only important for the diagnosis of ASFV, but may also provide targets for the development of new therapeutic strategies [13].

Common methods for preparing mAb include hybridoma technology, phage display technology, B cell immortalization technology, and single B cell antibody preparation technology. Currently, the single B-cell antibody preparation technique is the most efficient method for screening mAb with natural conformations, which could generate a large number of natural antibodies in a short period of time with a wide range of applications and have made important contributions to pathogen detection, antiviral therapy, and immune system research [14]. It has prepared the specific and sensitive mAbs for the diagnosis of various viruses with the expansion of immunized species from initially mice to other species such as rabbits and guinea pigs [15,16,17,18,19,20,21]. It has also significantly contributed to the development of large mammal-derived mAb. The natural porcine neutralizing antibodies have been generated from a single B cell of a CSFV C-strain E2-immunized pig [22]. The single B-cell antibody technology could be used to isolate and characterize the bovine-derived neutralizing monoclonal antibodies against FMDV serotype A [23]. A platform was established for the rapid screening of equine immunoglobulin fragment antigen binding F(ab)2 derived from single equine memory B cells [24].

Since there is no effective vaccine for ASF, the detection, prevention, and control of ASF are particularly important. Mabs can specifically recognize specific antigenic epitopes and have a wide range of applications in basic biological research, clinical diagnosis, and diagnostic treatment fields, such as targeted delivery of drugs and blocking of pathogen infection. Compared with traditional methods such as hybridoma technology, single B-cell antibody technology has the advantages of rapid, efficient, and high yield in antibody production, which also ensures the natural pairing of light and heavy chain variable regions. To date, hybridoma technology remains the dominant technique for the preparation of ASFV antibodies [25]. In this study, we established a platform for the rapid preparation of ASFV mouse-derived antibodies by single B-cell antibody technology, which efficiently and rapidly prepared mAb against ASFV CD2v protein and established a blocking ELISA method that can be used for the rapid clinical detection of ASFV CD2v protein to provide a powerful tool for monitoring ASFV in the field.

## 2. Materials and Methods

### 2.1. Virus, CD2v Protein, and Serum Samples

**Figure 1:**
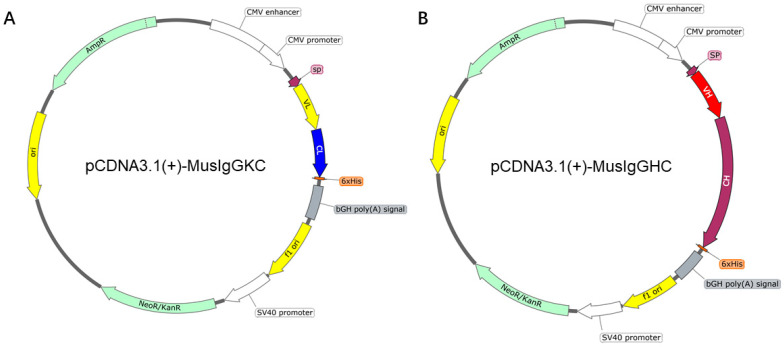
The plasmid profile of pCDNA3.1(+)-MusIgGHC and pCDNA3.1(+)-MusIgGKC expression vector. (**A**) The plasmid profile of pCDNA3.1(+)-MusIgGKC expression vector. (**B**) The plasmid profile of pCDNA3.1(+)-MusIgGHC expression vector.

ASFV-negative serum, ASFV-positive serum, Porcine reproductive and respiratory syndrome virus (PRRSV) positive serum, Classical swine fever virus (CSFV) positive serum, Porcine Circovirus 2 (PCV-2) positive serum, Porcine parvovirus (PPV) positive serum, ASFV-positive serum standards, ASFV genotype II strain, CD2v protein, pCDNA3.1(+)-MusIgGHC (Figure 1A), and pCDNA3.1(+)-MusIgGKC (Figure 1B) expression vector (IgG2) were all provided by the China Institute of Veterinary Drug Control, National/WOAH Reference Laboratory for Classical Swine Fever. The infection of the ASFV genotype II strain (HuB/HH/2019) in PAM cells was conducted in a biosafety level III laboratory of the IVDC, approved by the Ministry of Agriculture and Rural Affairs of the People’s Republic of China.

### 2.2. Mouse Vaccination and Single B-Cell Sorting by FACS

Three four-week-old BALB/c mice were inoculated with 60 µg of purified CD2v protein at 2-week intervals for a total of four inoculations. The blood for serum analysis was harvested from animals the day before the final boost vaccination. Splenocytes were harvested from animals 7 days following the final boost vaccination. For staining, freshly isolated splenocytes were stained with His-CD2v, BV421 anti-mouse CD21, PE-cy7 anti-mouse CD43, APC anti-mouse CD19, and Alexa Fluor 488 anti-mouse IgM antibodies (the above antibodies were purchased from BioLegend) for 20 min at 4 °C. The stained samples were immediately sorted by flow cytometry (BD FACS Aria II) using a 100 μm nozzle. CD2v-specific splenocytes were sorted for CD19^+^/CD21^+^/CD43^−^/IgM^−^/His-tag (CD2v^+^) events.

### 2.3. Amplification, Sequencing, and Expression of mAbs

After sorting, a Single Cell Full Length mRNA-Amplification Kit (Vazyme, Nanjing, China) was used for the amplification of cDNA immediately, according to the manufacturer’s instructions. The cDNA templates were stored at −20 °C for subsequent PCR amplification. For the amplification of antibody variable region genes, two seminested PCR amplifications (PCR primers are listed in Table 1 and Table 2) were run per well for the light and heavy chains. All PCRs were performed in 96-well plates in a total volume of 25 µL per well. The program of PCR was as follows: 98 °C for 3 min, followed by 45 cycles at 98 °C for 10 s, 60 °C for 15 s, and 72 °C for 25 s, and a final incubation step at 72 °C for 5 min. The paired heavy and light chain variable domain genes were inserted into the pCDNA3.1(+)-MusIgGHC and pCDNA3.1(+)-MusIgGKC expression vectors (Figure 1) separately for expression in HEK-293T cells and then purified from the culture supernatant to obtain antibodies at high concentration. The prepared monoclonal antibody was obtained from HEK-293T cell culture supernatant and purified by Protein G affinity chromatography. The purification column was first pre-equilibrated with PBS (pH 7.4) solution, and the culture supernatant filtered through a 0.45 µm membrane was loaded onto the chromatography column. Then, the antibody was eluted with 0.1 M glycine buffer (pH 2.7), and the eluate was immediately neutralized with 1 M Tris-HCl (pH 9.0) to protect the antibody activity. Purified antibodies were removed from impurities by dialysis (PBS, pH 7.4) and concentrated. Mabs were handed over to Beijing Tsingke Biotech Co., Ltd., Beijing, China for HRP labeling.

### 2.4. SDS–PAGE

A total of 2 μg of each antibody was subjected to a gradient 12% polyacrylamide gel. DTT (Solarbio) was added to the sample buffer with all samples incubated for 10 minutes at 95 °C. Novex™ Sharp Pre-stained Protein Standard (Invitrogen, Waltham, MA, USA) was used to assess the approximate size of proteins visualized by SolarFast SDS–PAGE Coomassie brilliant blue staining solution (Solarbio, Beijing, China).

### 2.5. Immunofluorescence Assay (IFA)

Porcine alveolar macrophage (PAM) cells seeded in plates (Corning, NY, USA) were infected with the ASFV at MOI = 1. After 36 h post-infection, the cells were fixed with precooled methanol-acetone solution (ratio 1:1, *v*/*v*) for 2 h at −20 °C and washed with PBS twice. The cells were incubated with different primary antibodies for 1 h at 37 °C. After rinsing with PBS three times, a 1:200 dilution of the FITC-conjugated goat-anti-mouse antibody (Thermo Fisher Scientific, Waltham, MA, USA) was added to each well for a 45 min incubation at 37 °C. Following three washes, the signal was visualized with a confocal laser scanning microscope (Leica, Wetzlar, Germany).

### 2.6. Indirect ELISA

The wells of ELISA plates were coated with 100 µL of purified CD2v protein diluted in coating buffers at 37 °C for 2 h. The plates were then thoroughly washed with TBST and blocked with 10% (*w*/*v*) nonfat milk in PBS at 37 °C for 2 h. After three washes with TBST, positive and negative control serum samples were diluted in PBS and transferred to the coated plates (100 µL per well), and the plates were incubated at 37 °C for 60 min. After three washes, 100 µL of goat anti-mouse IgG-HRP antibody (1:5000 dilution) was added to each well prior to incubation for 50 min at 37 °C. After washing, color was developed with 100 µL of TMB substrate at 37 °C for 10 min, and the reaction was terminated with 100 µL of stop solution. Finally, the absorbance at 450 nm (OD450) was measured using a Varioskan Lux instrument. Set the cutoff value at 2.1 times the average value of the negative control group. If the value exceeds the threshold, it is considered positive; if it does not exceed the threshold, it is considered negative.

### 2.7. Construction of Blocking ELISA

The wells of ELISA plates were coated with 100 µL of purified CD2v protein diluted in carbonate buffer at 37 °C for 2 h. The plates were then thoroughly washed with TBST and blocked with 10% (*w*/*v*) nonfat milk in PBS at 37 °C for 2 h. After three washes with TBST, positive and negative control serum samples were diluted (1:10 dilution) in PBS and transferred to the coated plates (100 µL per well), and the plates were incubated at 37 °C for 60 min. After three washes, 100 µL of HRP-conjugated monoclonal CD2v antibodies (1:100 dilution) was added to each well prior to incubation for 60 min at 37 °C. After washing, color was developed with 100 µL of TMB substrate at 37 °C for 10 min, and the reaction was terminated with 100 µL of stop solution. Finally, the absorbance at 450 nm (OD450) was measured using a Varioskan Lux instrument. After the optimum conditions were confirmed, swine serum samples with a known status were tested to evaluate the diagnostic performance of the mAbs. The sample results were recorded as percent inhibition (PI) values calculated using the following formula: PI  =  (OD450negtive − OD450sample) /OD450negtive × 100%.

## 3. Results

### 3.1. Specific Single B-Cell Sorting and Generation of mAbs Against CD2v

**Figure 2:**
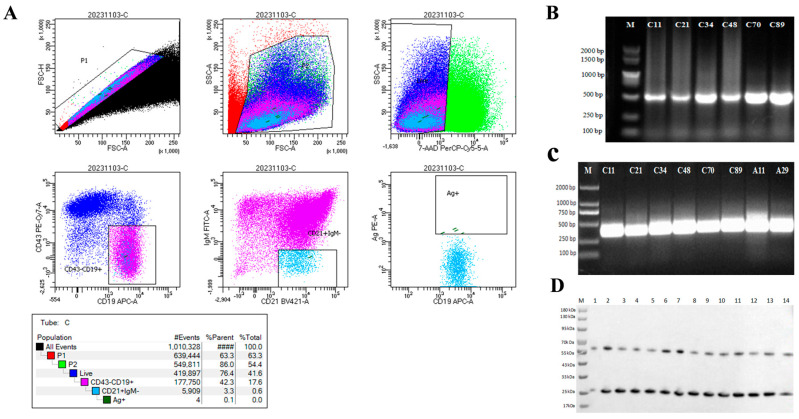
Sorting of CD2v-specific B cells and generation of mAbs against CD2v. (**A**) Mice PBMCs were stained with His-CD2v, BV421 anti-mouse CD21, PE-cy7 anti-mouse CD43, APC anti-mouse CD19, and Alexa Fluor 488 anti-mouse IgM antibodies for 20 min at 4 °C. The stained samples were immediately sorted by flow cytometry (BD FACS Aria II) using a 100 μm nozzle. CD2v-specific splenocytes were sorted for CD19^+^/CD21^+^/CD43^−^/IgM^−^/His-tag (CD2v^+^) events. (**B**) PCR results of partial antibody heavy chain variable regions. The specific bands appeared in the gel, with a size of approximately 500 base pairs. M: DNA Marker, C11, C21, C34, C48, C70, and C89 are single cell numbers. (**C**) PCR results of partial antibody light chain variable regions. The specific bands appeared in the gel, with a size of approximately 400 base pairs. M: DNA Marker, C11, C21, C34, C48, C70, and C89 are single cell numbers. (**D**) Western blot results of some Mabs. The image shows distinct double bands of 14 mAbs. One band is at around 60 kDa, while another band appears at approximately 26 kDa. M: protein marker, 1–14: Represent mAb C11, C14, C21, C24, C25, C28, C29, C32, C34, C48, C65, C66, C70, and C89, respectively.

To obtain ASFV CD2v-specific B cells, mononuclear cell populations were selected based on their size and granularity among all peripheral blood mononuclear cells (PBMCs). B-cell populations were specifically identified by markers CD19 and CD21, while T-cell populations were excluded by marker CD43 for negative selection. Subsequently, CD19^+^/CD21^+^/CD43^−^/IgM^−^/His-tag(CD2v)^+^ B cells were sorted by employing negative selection with the IgM marker and positive selection with the biotin marker (Figure 2A). ASFV CD2v-specific PBMCs isolated from splenocytes constituted approximately 0.1% of the cell population, with the acquisition of 96 ASFV CD2v-specific B cells. To obtain sequences that can express antibodies, we amplified the Variable heavy chain (VH) and Variable light chain (VL) and obtained a total of 22 sequences for the VH gene and 42 sequences for the VL gene. Among these sequences, 14 pairs of heavy-light chain matching sequences were successfully identified and expressed in HEK293 cells and then purified (Figure 2B–D).

### 3.2. Reactivity of mAbs by Indirect ELISA and IFA

**Figure 3:**
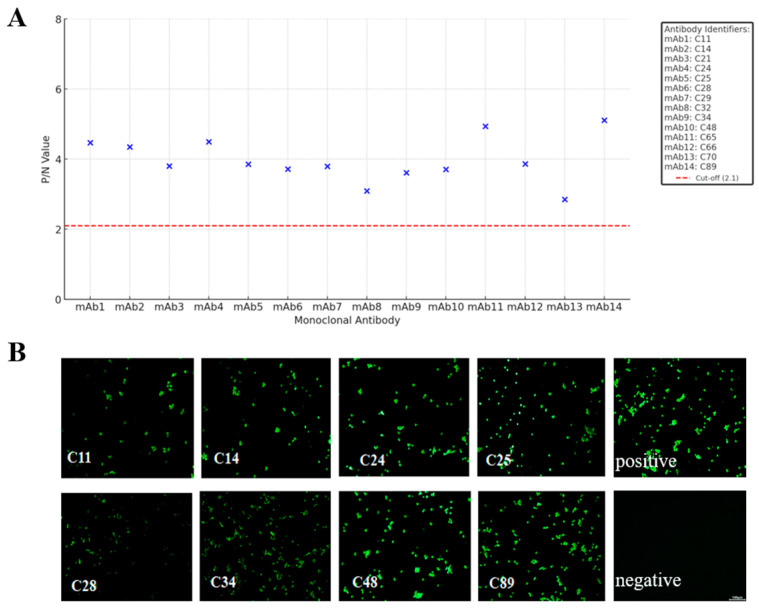
Reactivity of mAbs by indirect ELISA and IFA. (**A**) Indirect ELISA results of 14 mAbs. The wells of ELISA plates were coated with purified CD2v protein and goat anti-mouse IgG-HRP antibody. Set the cutoff value at 2.1 times the average value of the negative control group. If the value exceeds the threshold, it is considered positive; if it does not exceed the threshold, it is considered negative. (**B**) Reactivity of mAbs for IFA detection (10X). PAM cells were infected with the ASFV at MOI = 1. After 36 h post-infection, the cells were incubated with different mAbs and a FITC-conjugated goat-anti-mouse antibody. The signal was visualized with a confocal laser scanning microscope.

To validate the interaction of 14 mAbs with the CD2v protein, antibody affinity was determined by indirect ELISA (Figure 3A). Among the tested antibodies, all 14 mAbs demonstrated reactivity with the CD2v protein. To further verify that the mAbs react with the ASFV genotype II strain, we observed specific fluorescence of 14 mAbs with ASFV CD2v by IFA. IFA showed that 8 mAbs successfully detected ASFV CD2v in PAM cells (Figure 3B), indicating the tested mAbs can effectively recognize and bind to ASFV CD2v.

### 3.3. Construction and Standardization of Blocking ELISAs

A blocking ELISA method for detecting CD2v antibody against ASFV was initially established using recombinant CD2v protein as coating antigen, ASFV-positive serum and negative serum as positive and negative controls, and HRP-labeled CD2v mAb as blocking antibody (0.25 mg/mL). C89 mAb, with the highest P/N value and good reactivity in the IFA experiment, was selected as the blocking antibody to label HRP (Beijing Tsingke Biotech Co., Ltd.). In order to explore the optimal conditions of the blocking ELISA method, the optimum coating concentration, type of blocking solution, enzyme-labeled antibody dilution, incubation time of enzyme-labeled antibody, and color development time were determined using a checkerboard titration method (Tables S1–S5).

**Figure 4:**
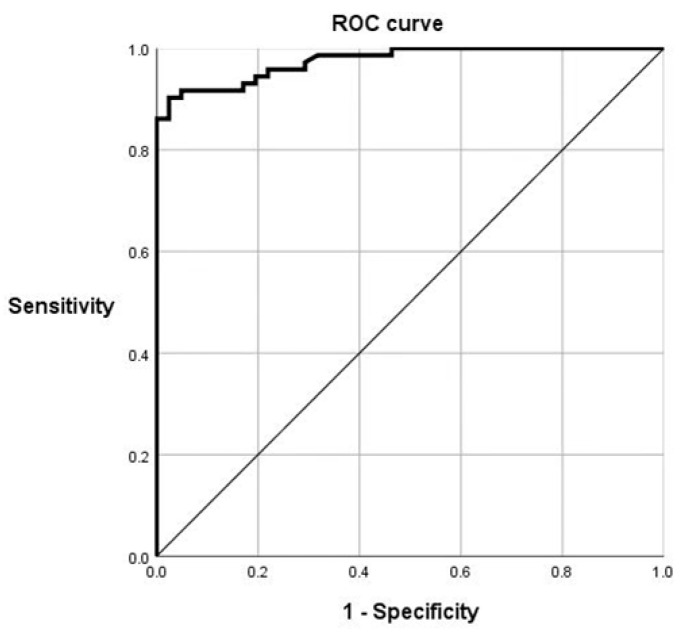
ROC curve analysis.

A total of 113 serum samples (41 negative serum samples and 72 positive serum samples provided by the China Institute of Veterinary Drug Control) with known status were used to estimate the diagnostic sensitivity (Dn) and specificity (Dp) of the C89 mAbs by receiver operating characteristic (ROC) curve analysis (Figure 4). The area under the ROC curve (AUC) is 0.975 with a standard error is 0.011, and the asymptotic 95% confidence interval is between 0.953 and 0.998, indicating a high accuracy in distinguishing positive and negative samples by the blocking ELISA method established in this study. The maximum Youden index of the ROC curve was 0.879, with the corresponding sensitivity values of 90.3%, specificity of 97.6%, and cut-off value of 51.0%.

### 3.4. Determination of the CD2v-Blocking ELISA Specificity, Sensitivity, Reproducibility, and Compliance Rate

**Figure 5:**
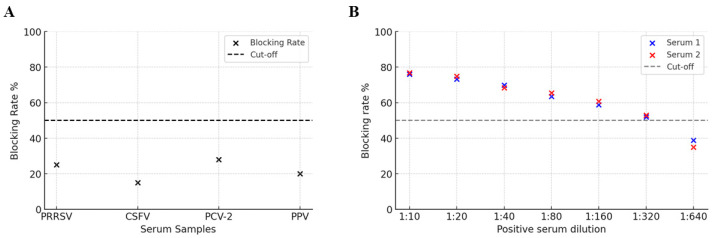
Determination of the CD2v-blocking ELISA specificity, sensitivity, reproducibility, and compliance rate. (**A**) The results of blocking ELISA specificity test. The CD2v-blocking ELISA did not detect PRRSV, CSFV, PCV-2, or PPV-positive serum samples at 1:320 dilution. (**B**) The results of blocking ELISA sensitivity assay. Three clinical ASFV-positive serum samples were diluted from 1:10 to 1:640 to determine the highest dilution of serum detected in the assay according to the cut-off value.

To determine the CD2v-blocking ELISA method specificity, we analyzed PRRSV, CSFV, PCV-2, and PPV-positive serum samples. The blocking rates were far below the negative threshold, which proved that the established blocking ELISA method exhibited excellent assay specificity (Figure 5A). To determine the CD2v-blocking ELISA sensitivity, we analyzed two clinical ASFV-positive serum samples diluted from 1:10 to 1:640 using the determined cut-off value of 51%. The highest dilution of the ASFV-positive sample that was detected in the assay was 1:320 (Figure 5B), indicating an assay sensitivity up to 1:320.

The reproducibility of the CD2v-blocking ELISA was determined based on intra- and inter-batch assays. The coefficient of variation (CV) values were calculated by analyzing five randomly selected ASFV-positive or ASFV-negative serum samples in a single batch and four different batches, respectively. As shown in Tables S6 and S7, the intra- and inter-assay values were both ≤7%, suggesting a high repeatability and low variability of the blocking ELISA.

To determine the CD2v-blocking ELISA compliance, we analyzed ASFV-positive (*n* = 62) and ASFV-negative (*n* = 38) serum samples tested by IFA. The assay showed 96.8% (60/62) compliance in the identification of ASFV-positive serum samples and 100% (38/38) compliance in the identification of ASFV-negative serum samples, while the total compliance rate was 98.0% (Table S8).

## 4. Discussion

In the past 100 years since the first discovery of ASFV, researchers have attempted to comprehensively characterize its pathogenesis, transmission mechanisms, and diagnostic methods, but still face many challenges. The functions and mechanisms of most proteins encoded by ASFV are not yet fully understood [26]. The lack of understanding of the interaction between ASFV and its host has hindered drug and vaccine development against ASFV [27]. In our research, the development of Mabs against ASFV is particularly critical, providing a powerful tool for further investigation of the underlying mechanisms of immune escape and host–virus interactions, as well as for vaccine development.

Single B-cell-based antibody platforms offer an effective approach for the discovery of useful antibodies for therapeutic or research purposes. Using the technology platform, it is also possible to isolate B cells from the organism at any point in time. With the maturity of high-throughput sequence amplification, second-generation sequencing, and other related technologies, it will facilitate the study of the entire immune system and its mechanisms, and provide a theoretical basis for the development of vaccines, drug therapy, and clinical research. The first critical step in the technology of single B-cell antibody preparation is the screening of individual-specific B cells. With technological advances, the means of screening B cells have become more abundant and efficient [28,29]. The widely applicable FACS method was selected as the means of obtaining specific B cells in this research. In determining the antibody screening conditions, this study chose to identify B cells by CD19 and CD21 labeling, while excluding T cells by CD43 labeling, and finally established the combination of CD19^+^/CD21^+^/CD43^−^/IgM^−^/His-tag^+^ as the screening conditions. Meanwhile, a single B-cell antibody technology platform for the preparation of ASFV murine-derived mAb was established. Fourteen strains of CD2v mAbs were successfully prepared using the prepared recombinant proteins as immunogens. Compared with traditional hybridoma technology, the mAb of other ASFV proteins can be rapidly prepared in a short period of time using this technology platform because of its higher efficiency, which can support basic research, vaccine development, and diagnostic reagent development of ASFV.

In addition, although the Mabs prepared in this study demonstrated good antigenic affinity and specificity (Table 3), the broad applicability and long-term effectiveness of these antibodies need to be further verified in the face of the high variability and complex transmission mechanism of ASFV. An indirect ELISA based on the CD2v protein has been established to identify wild-type and CD2v-deleted strains of ASFV at first [30]. To further differentiate wild-type and CD2v gene-deleted ASFV, a dual ELISA based on p30 and CD2v protein was established [12]. Another dual indirect ELISA based on p54 and CD2v proteins has been developed to specifically distinguish serum antibodies from pigs infected with wild-type ASFV or possessing attenuated vaccine immunization [31]. Besides the methods described above, the competitive ELISAs (cELISAs) based on the ASFV-p72 or ASFV-CD2v nanobody were developed to detect anti-ASFV antibodies, which could differentiate pigs infected with wild-type or CD2v-deleted ASFV [32]. Recently, a newly established triple protein-based ELISA could rapidly differentiate wild-type ASFV or CD2v and/or MGF505 gene-deleted strains infection [33].

The development of companion diagnostics using specific proteins of the virus is an effective way to realize the DIVA strategy [34,35]. The CD2v monoclonal antibody-based blocking ELISA developed in this study has significant potential applications in differentiating between infected and vaccine-immunized animal strategies, which is essential for effective control of ASFV. The ability to distinguish wild-type ASFV-infected animals from vaccinated animals has become particularly important as ASFV vaccine development has progressed, especially with attenuated strains constructed by deleting specific genes (e.g., CD2v). If a vaccine candidate is designed by deleting the CD2v gene, vaccinated animals will not produce anti-CD2v antibodies, whereas animals infected with wild-type ASFV will produce such antibodies. The detection of CD2v antibodies will enable the identification of animals infected with wild-type ASFV type II in the vaccinated population.

In summary, this study has made progress in the detection of ASFV using a single-B cell preparation platform, which demonstrated well for ASFVII detection. The performance parameters of this method need to be comprehensively evaluated to accurately calculate its diagnostic sensitivity and specificity by comparing it with existing gold standard diagnostic methods such as PCR and validated ELISA. However, further investigation is needed for the comprehensive control and prevention of ASFV, including the effect of virus mutation on the detection method, the performance validation of the detection method in practical applications, and the potential application of Mabs in viral mechanism research and vaccine development.

## Figures and Tables

**Table 1:** Primers for amplification IgG heavy chain variable region genes of murine.

Primers	Primer Sequence (5′ → 3′)
IgH-OF1	GGGGTGCAGCTGCAGGAGTC
IgH-OF2	GAGGTGCAGCTGCAGGAGTC
IgH-OF3	CAGGTTCAGCTCCAGCAGTC
IgH-OF4	CAGGTGCAGCTGAAGCAGTC
IgH-OF5	GAGGTTCAGCTGCAGCAGTC
IgH-OF6	GAGGTGCAGTTGGTGGAGTC
IgH-OF7	GAGGTTCAGCTGCAGGAGTC
IgH-OF8	GAGGTCCAGCTGCAACAGTC
IgH-OF9	GAGGTGCAGCTTCAGGAGTC
IgH-OF10	CAGATCCAGTTGGTGCAGTC
IgH-OF11	CAGATGCAGCTGCAGGAGTC
IgH-OR1	AGGGACTCTGGTCACTGTCTCTGCA
IgH-OR2	AGGGACCACGGTCACCGTCTCCTCA
IgH-OR3	AGGCACCACTCTCACAGTCTCCTCA
IgH-OR4	AGGAACCTCAGTCACCGTCTCCTCA

**Table 2:** Primers for amplification IgG light chain variable region genes of murine.

Primers	Primer Sequencea (5′ → 3′)
Igκ-OF1	CAAATTGTTCTCACCCAGTC
Igκ-OF2	GACATTGTGATGACCCAGTC
Igκ-OF3	GACATTGTGCTGACCCAATC
Igκ-OF4	GACATCCAGATGACACAGTC
Igκ-OF5	GACATCCAGATGACTCAGTC
Igκ-OF6	GATATCCAGATGACACAGAC
Igκ-OF7	GAAACAACTGTGACCCAGTC
Igκ-OF8	GATATTGTGATGACTCAGGC
Igκ-OF9	GATGTTTTGATGACCCAAAC
Igκ-OF10	GATGTTGTGATGACCCAAAC
Igκ-OF11	GACATCTTGCTGACTCAGTC
Igκ-OR1	GCTGGGACCAAGCTGGAGCTGAAA
Igκ-OR2	CGGCTCGGGGACAAAGTTGGAAATAAAA
Igκ-OR3	CGTTCGGAGGGGGGACCAAGCTGGAAATAAAA
Igκ-OR4	CGGTGGAGGCACCAAGCTGGAAATCAAA

**Table 3:** Reactivity of mAbs by IFA, Western blot, and indirect ELISA.

Sample Number	IFA	Western Blot	Indirect ELISA
	ASFVII		
C11	+	+	+
C14	+	+	+
C21	−	+	+
C24	+	+	+
C25	+	+	+
C28	+	+	+
C29	−	+	+
C32	−	+	+
C34	+	+	+
C48	+	+	+
C65	−	+	+
C66	−	+	+
C70	−	+	+
C89	+	+	+

+, positive; −, negative.

## Data Availability

Data is contained within the article or supplementary material. The original contributions presented in this study are included in the article/supplementary material. Further inquiries can be directed to the corresponding author.

## Supplementary Materials

The following supporting information can be downloaded at: https://www.mdpi.com/article/10.3390/v17081123/s1.
