# Supplementary material for: Development and Application of Mouse-Derived CD2v Monoclonal Antibodies Against African Swine Fever Virus from Single B Cells
Source: Viruses. 2025 Aug 15;17(8):1123. doi: 10.3390/v17081123 (PMC12390635; doi:10.3390/v17081123)
Supplement: Supplementary file 1 [file viruses-17-01123-s001.zip › viruses-3789443-supplementary.pdf]

viruses

MDPI

Supplementary data

Table S1. Determination of optimal antigen coating concentration.

Antigen coating concentration (μg/mL)	Positive serum OD450nm	Negative serum OD450nm	Blocking rate
4	1.047	3.249	67.8 %
2	0.852	3.208	73.4 %
1*	0.781	2.998	73.9 %
0.5	0.766	1.868	59.0 %
0.25	0.692	1.475	47.1 %

* Optimal conditions of the blocking ELISA method.

Table S2. Determination of the best blocking solution.

Blocking solution	Positive serum OD450nm	Negative serum OD450nm	Blocking rate
3 % BSA	0.991	2.934	66.2 %
5 % BSA	0.916	2.921	68.6 %
5 % nonfat milk	0.866	2.876	69.9 %
10 % nonfat milk *	0.745	2.881	74.1 %

* Optimal conditions of the blocking ELISA method.

Table S3. Determination of optimal enzyme-labeled secondary antibody dilution.

Dilution	Positive serum OD450nm	Negative serum OD450nm	Blocking rate
1:100*	0.856	3.185	73.1 %
1:500	0.774	2.823	72.6 %
1:1000	0.664	1.918	65.4 %
1:2000	0.538	1.312	59.0 %

* Optimal conditions of the blocking ELISA method.

Table S4. Determination of optimal enzyme-labeled secondary antibody incubation time.

Incubation time	Positive serum OD450nm	Negative serum OD450nm	Blocking rate
30 min	0.643	1.655	61.1 %
45 min	0.714	2.820	74.7 %
60 min*	0.712	2.968	76.0 %
75 min	0.810	2.982	72.8 %

* Optimal conditions of the blocking ELISA method.

Table S5. Determination of optimal substrate color development time.

color development time	Positive serum OD450nm	Negative serum OD450nm	Blocking rate
5 min	0.530	1.924	72.5 %
10 min*	0.717	3.051	76.5 %
15 min	0.957	3.216	70.2 %
20 min	1.461	3.219	54.6 %

* Optimal conditions of the blocking ELISA method.

Table S6. Intra-batch reproducibility testing.

Sample number	Blocking rate of 4 replicates in the intra-batch				Mean	SD	CV[a]
	1	2	3	4			
ASFV positive 1	68.2%	60.9%	69.1%	70.2%	67.1%	0.036	5.4%
ASFV positive 2	76.6%	70.2%	73.4%	78.0%	74.6%	0.030	4.0%
ASFV positive 3	71.2%	73.6%	72.4%	74.5%	73.0%	0.024	3.3%
ASFV negative 1	24.6%	23.2%	22.7%	23.8%	23.6%	0.007	3.0%
ASFV negative 2	25.7%	28.6%	26.7%	28.1%	27.2%	0.011	4.1%

[a]CV (coefficient of variation) = SD/Mean × 100%.

Table S7. Inter-batch reproducibility testing.

Sample number	Blocking rate of 4 replicates in the inter-batch				Mean	SD	CV[a]
	1	2	3	4			
ASFV positive 1	57.3%	64.2%	61.5%	69.6%	63.2%	0.045	7.0%
ASFV positive 2	58.6%	62.5%	65.1%	70.2%	64.1%	0.042	6.5%
ASFV positive 3	68.6%	75.4%	73.3%	76.4%	73.4%	0.030	4.1%
ASFV negative 1	22.6%	19.6%	23.4%	22.9%	22.1%	0.014	6.7%
ASFV negative 2	24.7%	22.6%	25.5%	27.4%	25.1%	0.017	6.8%

[a]CV (coefficient of variation) = SD/Mean × 100%.

Table S8. Compliance rate detection of sample.

Sample number	Blocking rate	IFA	Sample number	Blocking rate	IFA	Sample number	Blocking rate	IFA
PJ-05	1.9%	Negative	PJ-44	31.4%	Negative	PJ-49	39.4%	Negative
PJ-04	72.0%	Positive	PJ-46	7.6%	Negative	PJ-52	13.5%	Negative
PJ-07	68.2%	Positive	PJ-32	74.7%	Positive	PJ-55	11.8%	Negative
PJ-08	73.4%	Positive	PJ-33	70.2%	Positive	PJ-59	3.9%	Negative
PJ-02	54.9%	Positive	PJ-50	62.4%	Positive	PJ-60	-0.8%	Negative
PJ-10	75.6%	Positive	PJ-51	67.7%	Positive	PJ-61	43.8%	Negative
PJ-11	77.8%	Positive	PJ-34	77.6%	Positive	PJ-62	22.7%	Negative
PJ-03	41.6%	Positive	PJ-36	76.9%	Positive	PJ-64	46.2%	Negative
PJ-12	79.2%	Positive	PJ-37	70.7%	Positive	PJ-65	39.9%	Negative
PJ-13	81.0%	Positive	PJ-38	71.0%	Positive	PJ-66	-7.4%	Negative
PJ-17	75.8%	Positive	PJ-39	69.4%	Positive	PJ-67	2.2%	Negative
PJ-18	71.9%	Positive	PJ-72	54.4%	Positive	PJ-89	-8.3%	Negative
PJ-19	70.2%	Positive	PJ-43	78.9%	Positive	PJ-90	3.4%	Negative
PJ-20	84.8%	Positive	PJ-45	74.4%	Positive	PJ-91	2.4%	Negative
PJ-14	51.2%	Positive	PJ-47	71.5%	Positive	PJ-92	17.3%	Negative
PJ-21	75.9%	Positive	PJ-48	75.0%	Positive	PJ-93	8.4%	Negative
PJ-21	75.6%	Positive	PJ-53	76.8%	Positive	PJ-94	20.7%	Negative

Sample number	Blocking rate	IFA	Sample number	Blocking rate	IFA	Sample number	Blocking rate	IFA
PJ-23	68.7%	Positive	PJ-54	64.0%	Positive	PJ-95	44.8%	Negative
PJ-27	73.3%	Positive	PJ-57	71.3%	Positive	PJ-96	45.9%	Negative
PJ-29	83.4%	Positive	PJ-56	80.7%	Positive	PJ-97	32.1%	Negative
PJ-30	70.7%	Positive	PJ-58	72.2%	Positive	PJ-98	29.9%	Negative
PJ-31	82.7%	Positive	PJ-60	72.8%	Positive	PJ-99	24.1%	Negative
PJ-06	32.8%	Negative	PJ-75	47.4%	Positive	PJ-100	-4.4%	Negative
PJ-09	-2.2%	Negative	PJ-63	62.0%	Positive	PJ-79	80.6%	Positive
PJ-15	13.8%	Negative	PJ-68	81.8%	Positive	PJ-80	82.0%	Positive
PJ-16	19.7%	Negative	PJ-69	77.3%	Positive	PJ-81	83.7%	Positive
PJ-22	24.2%	Negative	PJ-70	70.3%	Positive	PJ-83	79.4%	Positive
PJ-25	14.0%	Negative	PJ-71	72.8%	Positive	PJ-84	80.6%	Positive
PJ-26	35.4%	Negative	PJ-73	52.0%	Positive	PJ-86	66.8%	Positive
PJ-28	9.0%	Negative	PJ-82	58.5%	Positive	PJ-85	61.4%	Positive
PJ-35	4.4%	Negative	PJ-74	82.0%	Positive	PJ-87	71.5%	Positive
PJ-40	-4.4%	Negative	PJ-76	81.8%	Positive	PJ-88	75.0%	Positive
PJ-41	18.3%	Negative	PJ-77	77.3%	Positive			
PJ-42	2.6%	Negative	PJ-78	70.3%	Positive			
